# Evaluating Equations for Predicting Enteric Methane Emissions in Dairy Cattle

**DOI:** 10.3390/ani16081270

**Published:** 2026-04-21

**Authors:** Fern T. Baker, Luke O’Grady, Martin J. Green

**Affiliations:** 1 School of Veterinary Medicine and Science, University of Nottingham, Sutton Bonington Campus, Loughborough, Leicestershire LE12 5RD, UK; luke.ogrady@ucd.ie (L.O.);; 2 Countryside and Community Research Institute, University of Gloucestershire, Francis Close Hall, Swindon Road, Cheltenham, Gloucestershire GL50 4AZ, UK; 3 Section of Herd Health and Animal Husbandry, School of Veterinary Medicine, University College Dublin, Stillorgan Road, D04 W6F6 Dublin, Ireland

**Keywords:** enteric methane prediction equations, enteric fermentation, neutral detergent fibre (NDF) and metabolised energy (ME)

## Abstract

Several prediction equations have been created, based on various dietary composition variables, to predict dairy cattle enteric methane emissions (EMEs). Inconsistencies in measuring EMEs have created difficulties comparing dairy cattle emissions between farms and inhibits certain in efforts to reduce emissions and work towards Net Zero. The aims of the current study were to gather existing EME prediction equations and evaluate the variability in their prediction results. An additional aim was to create a combined prediction equation, based on the dietary components with the highest predictive ability, representing the average prediction across existing equations, which accounted for the variation amongst existing equations. The 32 equations produced large variation in the prediction of EMEs for each of the 15 example diets, ranging from 12.49 to 34.27 g CH_4_/kg DM. To create a combined EME prediction equation, twelve combinations of dietary variables were evaluated using a mixed-effects model. An equation based on metabolised energy (ME) and neutral detergent fibre (NDF) was chosen (methane (CH_4_) = 0.33 × ME + 0.31 × NDF + 3.47), due to the significance of the predictor variables and low prediction error (RMSE = 1.47 g CH_4_/kg DM), with a random-effects residual variance of 2.32. The combined equation may act as a suitable compromise to compare emissions between studies accounting for unexplained variation.

## Simple Summary

To reduce the environmental impact of dairy cattle, we first need to measure their emissions, which are often predicted by equations. However, there are several equations to measure the emissions from dairy cattle, making it difficult to compare emissions across farms. A universal measure is needed. The current study gathered 32 prediction equations from the literature and, using 15 example dairy diets, shows the wide variety in emission outputs each generates. From this, the combined equation was created, using the dietary variables that predicted the emissions most accurately to form an “average” equation of existing equations that can be used universally. The combined equation was based on the energy and fibre content of the diet, as the dietary factors that predicted methane emissions most accurately. The combined equation may act as a suitable compromise to compare emissions between studies accounting for factors which cause variation in emissions, such as from differing cow types and their stage of lactation.

## 1. Introduction

Climate change is a substantial issue facing the world [1,2], caused by greenhouse gas (GHG) emissions emitted from human activity, such as methane (CH_4_), carbon dioxide (CO_2_), and nitrous oxide [3]. To tackle climate change and the issues it brings, research is needed to facilitate the reduction in emissions from human activity. One of these areas is the agricultural industry and livestock, particularly cattle, as globally they account for 65% of emissions from the livestock sector in agriculture [4], due to enteric fermentation (EF) of feed [5]. EF creates methane as a byproduct [6], which has a global warming potential (GWP) between 28 and 34 times higher than carbon dioxide over 100 years [7] and is the main anthropogenic cause of methane emissions globally [8].

Various methods can be used to measure enteric methane emissions (EMEs) directly from livestock, such as the respiratory chamber, sulphur hexafluoride (SF_6_) tracer gas technique, ventilated hood, and the greenfeed system. Respiratory chambers are considered the most accurate measurement of EF emissions [9,10]. Measuring EF emissions directly is time consuming and requires expensive specialised technology and trained individuals to ensure reliability [9,10,11]. Often, these measurements are used to develop prediction equations of EMEs, utilising general linear models to predict emissions based on feed intake and dietary variables.

EME prediction equations vary in complexity, with most using simple readily available values, such as gross energy, while more complex equations include several dietary composition variables [10,12]. The IPCC [13,14] Tier 2 equations based on GEI are often favoured in models but were built for use as a national inventory and not as a tool to predict an individual farm’s emissions and evaluate mitigation strategies [15]. A review found that DMI only equations were approximate to complex equations in their results and could be used for record reporting [10]. However, DMI and GEI only equations do not account for the effect of dietary variables on EMEs or the comparison of diets on EMEs [16]. The fibre, fat, and protein content of feed have been shown to influence EMEs [17,18,19,20,21,22,23,24], demonstrating a need to consider possible trade-offs between model complexity and accuracy in equation development [10].

In their review, Appuhamy et al. [25] assessed the performance of prediction equations for representing the EMEs of cattle for different regions and ranked them based on root mean square prediction error (RMSPE) and concordance correlation coefficient. Ten of the twenty-two top ranking equations included at least one other factor, besides dry matter intake (DMI) or gross energy intake (GEI), with five containing at least three factors. The lack of agreement between equations is perhaps unsurprising, given they are not commonly developed and compared across multiple diets, with most based on emissions data from a total mixed ration diet, or limited concentrate or forage intake [10]. There is also the potential for many sources of variability between these studies, including measurement methods, dietary ingredients, study population size, region, and farming system.

Further research is needed into the degree of variation beyond simple measurements of dietary composition, using a large selection of diets [10]. There are also unexplained sources of variability in methane emissions, (e.g., cow type, their stage of lactation, etc.) which are not regarded in most published prediction equations, raising further concerns regarding their generalisability.

Previous reviews have not evaluated the degree of variation in predicted methane emissions between enteric equations or assessed which dietary composition variables within the prediction equations have the greatest influence. The aims of the study were to use example diets to explore variability between published EME equations, their predictions, which include dietary composition variables, and to use the predicted values from the range of equations to create a combined prediction equation accounting for the variability between published studies.

## 2. Materials and Methods

### 2.1. Sourcing Equations

Equations predicting emissions from EF were identified using the databases Science Direct, Google Scholar and PubMed, by searching for peer-reviewed scientific publications in English. The terms “dairy cattle”, “prediction”, “equation(s)”, “methane” and “enteric fermentation emissions” were searched, which, after removing duplicates, resulted in 239 papers. The article titles and abstracts were screened to remove those irrelevant, before they were examined for equations predicting EMEs from dairy cattle. This resulted in 24 articles being selected, from which the prediction equations were extracted, forming 132 enteric equations. The equations were coded as functions into R statistical programming language, version 4.2.2 [26]. Some equation outputs were reported as megajoules per day, which were converted to the same functional unit of methane (CH_4_) grams per day (CH_4_ g/day), by dividing methane emissions by 0.05565. The energy content of methane is equivalent to 55.65 kJ/g CH_4_, which means that 0.05565 megajoules of energy is equivalent to one gram of methane [25,27,28]. To allow assessment between diets with differing DMI, the results were divided by DMI, to produce methane emissions as grams per kg of DM.

### 2.2. Refining the Equations

**Figure 1:**
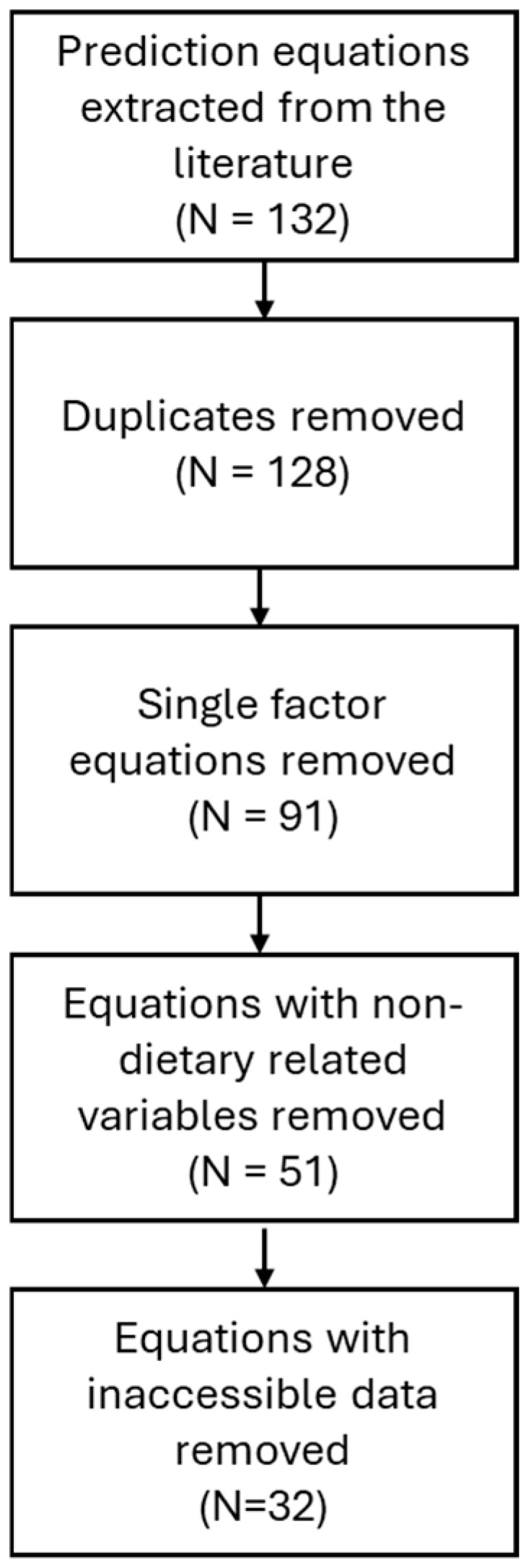
The exclusion process for the enteric methane prediction equations. N = the number of equations remaining after each exclusion step.

To facilitate the creation of the combined equation for dairy cattle accounting for the variability between published studies, the equations were refined by removing duplicates (*n* = 4) and simplistic equations based on only DMI or GEI (*n* = 37), as they do not account for dietary composition factors, which is essential for distinguishing between nutritional strategies. Non-dietary related variables were also excluded, such as milk yield, milk fat and milk protein content (*n* = 40). Equations with inaccessible or overly complex data requirements inhibiting reliable use, such as the digestibility of the dietary composition variables, fatty acid breakdown (C18:0), or those created for one specific diet, were excluded (*n* = 19). The exclusion process can be seen in Figure 1, which resulted in 32 remaining enteric equations from 5 papers, as shown in Table 1, as originally presented in the project report [29].

### 2.3. Defining Diets

In consultation with a specialist dairy cow nutritionist, the authors complied a dataset of 15 individual dairy cow diets, commonly used in UK commercial dairy farms. The choice of diets was intended to be representative of different cow stages of production, milk yield, production system types and seasons common in the United Kingdom and Europe, as well as some more extreme variations in dietary compositions. Diets 1–13 were for lactating dairy cows, while diets 14 and 15 were for dry cows, to represent real-world ration diversity for the whole animal cycle. Nutrition values for each dietary component were sourced from the reference feed values, from the feed into milk database [34].

### 2.4. Descriptive Analysis Model EME Predictions

The prediction equation was created utilising dietary variables from the 15 dairy diets, which were used as inputs for the 32 prediction equations, resulting in 480 EME predictions (32 equations applied to 15 diets). Table 2 shows the scope of emissions per diet. The 32 equations included the variables NDF, MEI, DMI, ADF, FA, CP, EE, percentage of forage and ash, as shown in Table 1, but the relevant dietary composition variables incorporated in the models were NDF, ADF, ME, GE, DM, FE, EE, and CP. The equations did not include variables that described the silage fermentation characteristics, such as organic acid profile or silage pH.

The results were plotted utilising “ggplot2” (v3.4.0) in R (v4.2.2) and initially assessed graphically. The distributional characteristics of emissions for each equation were determined. A correlation matrix was established through “corrplot” (v 0.92) in R, to explore the correlations between the dietary components, using the fifteen formulated diets. The correlations between the dietary variables were analysed to avoid bias in the equation, as seen in Table A1.

### 2.5. Evaluating the Variability Between EME Equations

The variability in emissions between equations was examined using a mixed linear regression model. The 32 equations applied to 15 diets, leading to a set of 480 EME predictions, were examined by applying the R programming language package “lme4” (v1.1-31) [26]. This structure created clustered data, with predictions nested within equations. To account for this dependency, a random-intercept structure was used, with prediction equation specified as a study-level random effect. Random intercepts were selected because exploratory model fitting showed that allowing slopes to vary by equation resulted in non-convergence and no substantive improvement in model performance. Using random intercepts created a unified predictive model that reflected shared biological patterns across studies.

Data were centred and standardised to aid model convergence [35] using the scale() function in R, such that

$$X_{j}^{*}=\frac{X_{j}-{\overset{ˉ}{X}}_{j}}{SD(X_{j})},$$

where $X_{j}$ is the original predictor, $X_{j}^{*}$ is the standardised predictor, ${\overset{ˉ}{X}}_{j}$ is the sample mean and $SD(X_{j})$ is the sample standard deviation of predictor j.

The mixed-effects model was fitted using these standardised predictors.

For each predictor $X_{j}$, the unstandardised regression coefficient was obtained by reversing the scaling operation. Because scale() divides the centred predictor by its standard deviation, the correct back-transformation for the slope is

$$\beta_{j,\mathrm{raw}}=\frac{\beta_{j}^{*}}{SD(X_{j})}.$$

where $\beta_{j}^{*}$ is the coefficient from the standardised model. This rescales the standardised slope $\beta_{j}^{*}$ back into the units of the original dietary variable.

To recover the intercept on the original scale, the means of the unscaled predictors must be reintroduced:

$$\beta_{0,\mathrm{raw}}=\beta_{0}^{*}-\sum_{j}\beta_{j}^{*}\left(\frac{{\overset{ˉ}{X}}_{j}}{SD(X_{j})}\right),$$

where $\beta_{0}^{*}$ is the intercept from the standardised model. This ensures that when all predictors take their original mean values, the model yields the correct methane prediction on the raw scale.

Where highly correlated variables, such as NDF and ADF (1.00), GE and ME (1.00), GE/ME and EE (0.86), and GE/ME and CP (0.77), were identified, only one dietary characteristic was considered for inclusion in the models. All combinations of the remaining variables were considered, and twelve final equations of different dietary composition constituents were chosen: NDF, GE, ME, FA, CP, and EE, which are shown in Table 3 below. Each equation included either GE or ME, as a feed-intake-related proxy in the equation, as suggested by Hristov (2018) [10]. The twelve equations from Table 3 were each individually coded into the R programming software to analyse their performance. The eight nutritional values in the twelve models were univariately screened for significant statistics and low error.

### 2.6. Assessment of Model Performance

The performance of the twelve equations were analysed against the variation in the published equations and the fifteen diets, using boxplots formulated utilising the “ggplot2” package in R [26]. The results of the combined prediction equation were compared to the median and range of the published equations. The boxplots, fixed-effects results, r^2^, root mean square error and residuals of variation were assessed to support which dietary composition equation was selected for the final combined prediction equation. The chosen mixed-effects linear regression model was fitted using metabolisable energy (ME) and neutral detergent fibre (NDF) as fixed effects, with prediction equation included as a random intercept. The model can be written as

$$y_{ij}=\beta_{0}+\beta_{1}{\mathrm{ME}}_{ij}+\beta_{2}{\mathrm{NDF}}_{ij}+u_{i}+\varepsilon_{ij},$$

where $u_{i}∼N\left(0,\sigma_{\mathrm{equation}}^{2}\right)$ and $\varepsilon_{ij}∼N(0,\sigma^{2})$, where $y_{ij}$ is the methane emission (g/kg DM) predicted by equation i for diet j; ${\mathrm{ME}}_{ij}$ is metabolizable energy (MJ/kg DM); ${\mathrm{NDF}}_{ij}$ is neutral detergent fibre (g/100 g DM); β_0_, β_1_, β_2_ are fixed-effects parameters; $u_{i}$ is a random intercept for prediction equation i with $u_{i}∼N\left(0,\sigma_{\mathrm{equation}}^{2}\right)$; and $\varepsilon_{ij}∼N(0,\sigma^{2})$ is the residual error term.

Degrees of freedom for fixed effects were computed using Satterthwaite’s approximation as implemented in the *lmerTest* package (v1.1-31). The model was fitted to 480 observations nested within 32 prediction equations. These values represent deterministic outputs from published equations rather than independent experimental observations. Within each diet, the 32 predictions share identical dietary ME and NDF values and, therefore, do not provide independent information on the fixed effects. Instead, the effective information for estimating the ME–NDF–methane relationship arises from the 15 diets, while the equation-level replication informs the random-intercept variance associated with between-equation heterogeneity. Collinearity among fixed-effects predictors was assessed using variance inflation factors (VIFs). Both ME and NDF had VIF values of 1.87, indicating low collinearity and meeting recommended thresholds for mixed-effects modelling for under 2.5 [36]. Building on previous work [29], the combined equation was evaluated using a leave-one-diet-out cross-validation and a sensitivity analysis which entailed the in-turn removal of each equation and the mixed-effects model refitted.

## 3. Results

### 3.1. Diet Composition

A summary of the fifteen formulated diets used in the equations can be seen below in Table 4.

### 3.2. Analysing the Variability Between Enteric Equations

**Figure 2:**
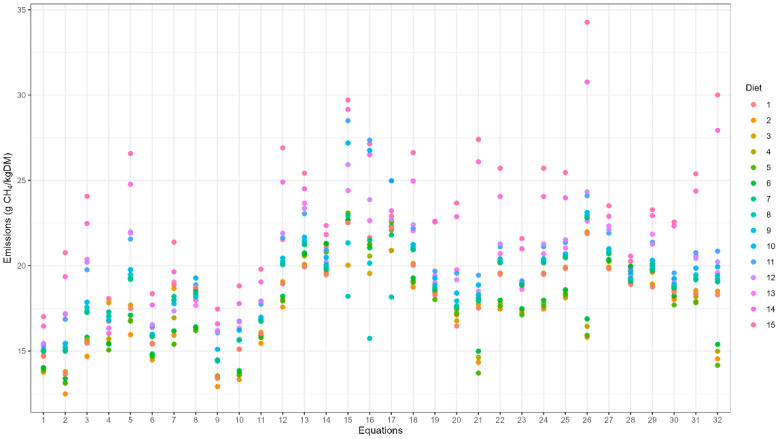
The enteric methane emissions (g CH_4_/kg DM) utilising the 15 dairy diets across the 32 prediction equations.

The final set of 32 published EME prediction equations utilized in the study showed variability in their predicted methane emissions, even when the same inputs were used, as shown in Figure 2. The results from the 32 equations reached from 12.49 to 34.27 g CH_4_/kg DM and reflected the effect of dietary composition on EMEs.

**Figure 3:**
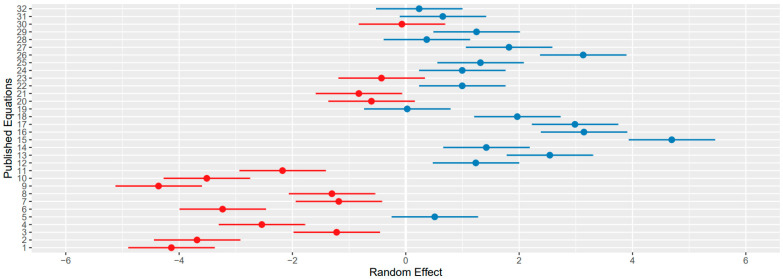
The variation in random effects for the 32 selected enteric prediction equations. Blue = positive random effect values (above 0), red = negative random effect values (below 0).

Figure 3 showcases the dispersal of random effects for the refined equations incorporated into the final combined equation. The figure highlights the range between equations within the same paper.

### 3.3. Choosing the Combined Prediction Equation Dietary Variables

There were strong correlations between the variables GE/ME and EE (0.86), and GE/ME and CP (0.77), which would bias the results if used in the same equation, limiting their usage.

### 3.4. Analysing the Performance of the Equations

There were twelve possible combinations of dietary variables for use in the combined equation, and the performance of each of the twelve equations were assessed based on their R^2^, root mean square error (RMSE) and residual variance, as shown in Table 5. The fixed-effects model showed significant results for all the variables included in an equation in four of the tested combined prediction equations, namely, equation 1: ME and NDF, equation 2: gross energy (GE) and NDF, equation 4: ME, NDF and FA, and equation 8: GE, NDF and FA, seen in Table 5. The results were similar between the models, but, based on the model performance, variation in results, error, significant t-values, and simplicity, ME and NDF were deemed suitable covariates to represent the combined prediction equation. The parameters were back-transformed from the centred and standardized values seen in Table 5 to the unstandardized combined equation (equation one) based on the raw diet data:

$$Methane\;({CH}_{4})=0.33*ME+0.31*NDF+3.47$$

Methane = grams of CH_4_ per kilogram of dietary dry matter intake (g/CH_4_/kg DM); ME = metabolized energy concentration expressed as megajoules per kilogram of dry matter (MJ/kg DM); NDF = fibre concentration of the diet expressed as grams of NDF per 100 g of dietary dry matter (percentage of DM).

**Figure 4:**
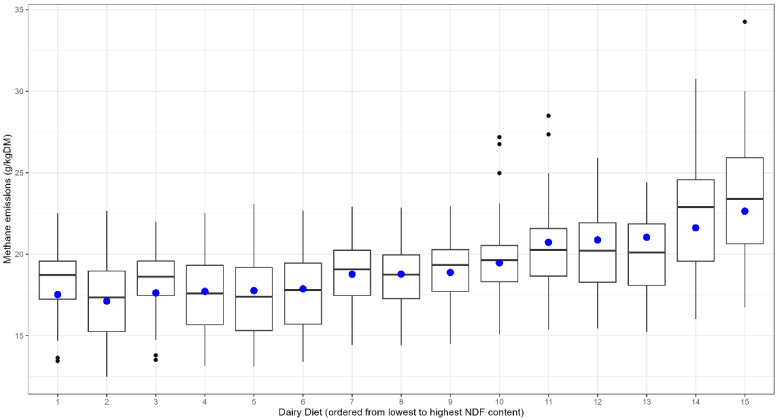
The blue dots represent enteric methane emission (EME) predictions for 15 realistic diets from the combined prediction equation, based on model 1 (mixed-effects model utilizing metabolised energy and neutral detergent fibre). The boxplots represent the distribution in predictions from 32 previously published EME predictions. Diets are presented from lowest to highest NDF content.

The performance of the chosen model based on ME and NDF was evaluated against the 15 diets and variability between the 480 EME results, which can be seen in Figure 4. The combined equation was also evaluated using a leave-one-diet-out cross-validation, which demonstrated good generalisation performance when trained on 14 diets and tested on the held-out diet. Cross-validated R^2^ values ranged from 0.25 to 0.96 across diets, with a mean R^2^ of 0.66 and median R^2^ of 0.67. The lowest R^2^ values were observed for diets 8 (R^2^ = 0.25), 15 (0.36), and 14 (0.48). Corresponding RMSE values ranged from 0.46 to 3.23, with a mean RMSE of 1.50. To assess whether the combined equation was driven by any single published equation, we performed a sensitivity analysis where each equation was removed in turn and the mixed-effects model refitted. The fixed-effects coefficients remained highly stable across runs (ME coefficient range: 0.20–0.43; NDF coefficient range: 0.28–0.32). This indicates that the combined methane prediction equation is not overly influenced by any single published equation.

## 4. Discussion

The study compiled and evaluated the variability between enteric methane prediction equations, utilising dietary composition and intake. Applying the same dietary composition values, the equations produced large variations in their methane emission predictions, demonstrating the uncertainty when comparing research on cattle emissions and creating mitigation strategies. A random-effects model and a range of example diets were used to model the predictions from each equation. A combined EME prediction equation was defined, accounting for the dietary proportions of energy and fibre and the unexplained sources of variability from numerous studies, used to generate the predictions. The combined equation facilitates easier comparison between studies and diets when developing mitigation strategies to reduce methane emissions from cattle.

The EME results from the 32 prediction equations ranged from 12.49 to 34.27 g CH_4_/kg DM, which could have been due to the varying methodologies between studies when creating the equations, which Hristov et al. [10] highlighted in their review of prediction equations. The studies differed in their country of origin and size, from small-scale studies of 16 cow records to worldwide meta-analyses of over 5000 cow records. The method of collection also varied for the EMEs, from respiration chambers to more variable methods, such as the SF_6_ technique, which varies by between 5–10% (approximately 14.3 to 29.1 g CH_4_/day) when compared to respiratory chamber emissions [9]. Importantly, some of the studies included a variety of livestock, which may vary considerably in their emissions [12]. The large variation creates huge uncertainty when attempting to predict enteric methane emissions for comparison across farms and between diets to accurately develop mitigation strategies, which the combined equation overcomes. The equations used in the current study were refined and only included those that included dairy and beef cattle.

The random-effects variance quantified the remaining variability between prediction equations after accounting for dietary ME and NDF effects. This variance reflects systematic differences among the 32 published equations arising from factors not explicitly modelled in the combined equation. These differences include biological and experimental factors such as variation in animal type and physiological state, such as breed, body weight, and lactation stage, as well as methane measurement method from respiration chambers, SF_6_, to the greenfeed system, and the specific mathematical assumptions embedded within each published equation. The partitioning of this variation was not conducted in this study and would have been difficult to account for.

Practically, this variance indicates that even when diets have identical ME and NDF values, the 32 published equations do not fully agree on their baseline methane output. The random-effects term, therefore, captures the unexplained structural heterogeneity among equations. Users of the combined equation should interpret this as the expected between-equation uncertainty: the combined equation predicts the mean relationship across studies, but individual predictions may vary by approximately ±√2.32 ≈ 1.52 units purely due to equation-level differences not attributable to diet composition.

Importantly, this variance does not reflect error in the combined equation itself but, rather, the inherent diversity of the underlying literature. It quantifies the real-world uncertainty associated with applying prediction equations across experiments, measurement systems, and animal populations and the variability expected when applying any methane equation to new diets. The combined equation highlights the value of the mixed-effects approach for generating a generalisable mean prediction across heterogeneous sources.

The combined equation derived in the study synthesises the results from 32 existing equations drawn from 5 published articles, which may limit the generalisability of the results. However as previously stated, existing equations derived from single studies are restricted, as they do not account for between-study or between-equation heterogeneity. The study followed a strict exclusion criterion to mitigate this, when refining the equations (see Figure 1), to improve accuracy and robustness of predictions. The combined equation was developed using 15 UK-based dairy diets, each applied across the 32 published methane prediction equations, yielding 480 equation-driven methane predictions for model fitting. Although the mixed-effects model was fitted to this full set of predictions, these values represent deterministic model outputs rather than independent biological observations. Consequently, the effective sample size for estimating fixed effects corresponds to the number of unique diets, while replication across equations informs the random-intercept variance associated with between-equation heterogeneity.

As a result, uncertainty in the ME and NDF coefficients reflects dietary coverage rather than equation count, and the fixed-effects estimates should be interpreted as representing the average relationship across a limited but diverse set of diets. Importantly, the mixed-effects framework enables a clear separation between diet-level information driving fixed-effects estimation and equation-level variability captured by the random effects, yielding stable and interpretable coefficient estimates despite the modest number of distinct diets. This interpretation is supported by the collinearity diagnostics and the consistency of cross-validation performance across the majority of diets.

The leave-out-one-diet cross-validation demonstrated good generalisation performance, and the sensitivity analysis revealed that no single prediction equation dominated the combined equation. The lowest cross-validated R^2^ value (0.25) was obtained for diet 8, which was a high-acid grass silage diet. Such diets are characterised by elevated concentrations of fermentation acids and altered carbohydrate fermentability, factors that are not explicitly represented by ME or NDF. These attributes can substantially influence rumen fermentation pathways and methane production, leading to increased divergence among published prediction equations. Consequently, a larger proportion of between-equation variability remains unexplained for this diet. While diets 14 and 15 corresponded to dry cow diets characterised by very high forage and NDF contents and low metabolisable energy intake, these diets lie outside the core dietary range of lactating cow systems for which most published methane equations were developed. Consequently, disagreement among equations is larger under dry cow conditions, and a greater proportion of variance remains unexplained by ME and NDF alone.

The inclusion of two dry cow diets in the formulation of the combined equation broadens the range of feeding systems represented and allows methane outputs to be evaluated across dietary contexts encountered in commercial practice. However, the combined equation primarily reflects lactating cow feeding systems, and predictions for dry cows should, therefore, be interpreted with caution due to fundamental differences in intake level, forage proportion, and rumen fermentation dynamics that are not fully captured by ME and NDF alone. Consequently, the wide range of diet-specific R^2^ values arises primarily for diets with atypical fermentation characteristics or at the boundaries of the predictor space. Importantly, the median R^2^ (0.67) was similar to the mean R^2^ (0.66), indicating that overall model performance was not driven by a small number of well-predicted diets but reflects consistent predictive ability across the majority of lactating cow diets.

The published equations included a variety of dietary predictor variables, but demonstrated consistency in their ranking, to reflect differences in dietary nutritional composition. The combined equation defined in the study is consistent with previous findings in the literature, highlighting the importance of energy and fibre content in influencing EMEs [17,19,20]. The relationship between the fibre content and EMEs is clearly captured in the combined equation, where the diet EMEs increased with the NDF content of the diets (see Figure 4). This demonstrates that fibre is influencing the model’s methane response, consistent with known digestive physiology, capturing the gradient across diets. However, the final model did not contain the effects of fat and protein content on EME predictions [17,18,19,20,21,22,23,24], which could have been due to the high correlation between the variables GE and ME with EE (0.86) and CP (0.77), commonly observed in the diets used in this study, limiting the use of these combinations of factors in the creation of prediction equations. Benaouda et al. [12] reviewed the performance of 40 dairy cattle enteric methane predictions and found that increased fat content did not decrease EMEs, reporting that diets with higher EE had a larger DMI and NDF content than the lower-EE diets. However, the authors acknowledge that the exclusion of EE does not negate the potential role of fat in mitigation strategies but reflects collinearity in the dataset.

To minimise the impact of correlations of dietary composition with DMI, predictions were evaluated based on grams of methane per kilogram of dry matter, as suggested by Benaouda et al. [12]. Only three prediction equations included FA, suggesting a small influence on the accuracy of the prediction equation if included. The selection of the most parsimonious model containing ME and NDF was considered the most influential and in agreement with Hristov et al. [10], who stated that the trade-off between model complexity and prediction accuracy should be considered when creating the equation. Niu et al. [28] also recommended the simple equation of DMI and NDF, compared to the better performing equation that included five variables based on DMI, NDF, EE, BW, and milk fat. Niu et al. [28] recommended the former due to its simplicity and ease of access to the required data compared to the best performing equation, which required knowledge of five variables to function. Previous research by Ellis et al. [30] found DMI superior to MEI in predicting EMEs, with lower RMSPE and greater R^2^ values. However, DMI equations do not allow comparison of dietary composition variables on EMEs [16], due to the strong correlation between DMI and EMEs that could suppress the effect of dietary variables on EMEs [12], which the current study aimed to capture.

The authors acknowledge that the exclusion of DMI to isolate the effect of dietary composition may introduce potential loss of predictive reliability, but MEI was used as a feed intake proxy to mitigate this loss. The exclusion of DMI also has practical utility, as DMI is often not known in advance, or requires estimation. Thus, the combined equation supports early-stage diet screening, allowing methane estimation prior to DMI measurements, and excludes potential error from DMI estimation. Further research can extend the verification of the current results by using independent animal-level data against the combined equation.

The authors also acknowledge that the diets represent primarily UK dairy production systems; therefore, the range of ME and NDF values reflects temperate feeding systems. As such, extrapolation to diets from markedly different regions, such as tropical forages, or maize-based total mixed rations with different fibre chemistry, should be made with caution. Future work incorporating diets from a wider range of production systems and regions would strengthen the global applicability of the combined equation.

## 5. Conclusions

The existing 32 EME prediction equations evaluated showed a range in their prediction results from 12.49 to 34.27 g CH_4_/kg DM, a 21.78 g CH_4_/kg DM spread representing 174% variation around the median. The large variation causes difficulty when generalising the results of any individual equation. The current study created a combined prediction equation representing the average prediction across 32 published equations and 15 dairy diets, based on dietary components ME and NDF. The combined equation utilised dietary characteristics derived in the study and provides a less-biased approach to accommodate the range of predictions. The combined equation may operate as a compromise solution, allowing easier comparison between studies, until further research establishes new factors or improved measurement methods that allow greater accuracy in predicting the impact of dietary variables on EMEs.

## Figures and Tables

**Table 1:** The 32 enteric methane prediction equations used in the model and the authors they were created by. NDF = neutral detergent fibre, MEI = metabolised energy intake, ADF = acid detergent fibre, EE = ether extract, FA = fatty acids, CP = crude protein [29].

Author	Model	Prediction Equation
Ellis et al. [30]	4c	CH_4_ g/day = (4.42 + 1.58 × NDF)/0.05565
	5c	CH_4_ g/day = (1.70 + 0.0667 × MEI + 0.0314 × Forage)/0.05565
	6c	CH_4_ g/day = (3.44 + 0.502 × DMI + 0.506 × NDF)/0.05565
	7c	CH_4_ g/day = (3.63 + 0.0549 × MEI + 0.606 × ADF)/0.05565
	8c	CH_4_ g/day = (4.41 + 0.0224 × MEI + 0.980 × NDF)/0.05565
	10c	CH_4_ g/day = (3.41 + 0.520 × DMI − 0.996 × ADF + 1.15 × NDF)/0.05565
	4d	CH_4_ g/day = (3.14 + 2.11 × NDF)/0.05565
	5d	CH_4_ g/day = (5.87 + 2.43 × ADF)/0.05565
	6d	CH_4_ g/day = (1.21 + 0.0588 × MEI + 0.0926 × Forage)/0.05565
	7d	CH_4_ g/day = 1.64 + 0.396 × MEI + 1.45 × NDF/0.05565
	8d	CH_4_ g/day = (2.16 + 0.493 × DMI − 1.36 × ADF + 1.97 × NDF)/0.05565
Van Lingen et al. [31]	1	CH_4_ g/day = -48.5 + 13.9 × DMI + 5.22 × ADF
	3	CH_4_ g/day = (11.0 + 0.335 × ADF) × DMI
Moate et al. [32]	2	CH_4_ g/day = [24.51 − 0.788 × EE] × DMI
Nielsen et al. [33]	1	CH_4_ g/day = (1.36 × DMI − 1.25 × FA − 0.20 × CP + 0.170 × NDF)/0.05565
	2	CH_4_ g/day = (1.23 × DMI − 1.45 × FA + 0.120 × NDF)/0.05565
	4	CH_4_ g/day = (1.39 × DMI − 0.91 × FA)/0.05565
Niu et al. [28]	3	CH_4_ g/day = 33.2 + 13.6 × DMI + 2.43 × NDF
	4	CH_4_ g/day = (163 + 13.3 × DMI − 11 × EE)
	5	CH_4_ g/day = 76 + 13.5 × DMI − 9.55 × EE + 2.24 × NDF
	6	CH_4_ g/day = 369 − 14.7 × EE + 1.67 × NDF
	16	CH_4_ g/day = -26 + 15.3 × DMI + 3.42 × NDF
	17	CH_4_ g/day = 160 + 14.2 × DMI − 13.5 × EE
	18	CH_4_ g/day = 11.3 + 14.7 × DMI + 2.5 × CP − 10.8 × EE + 3.2 × NDF − 2.87 × ash
	19	CH_4_ g/day = 435 − 18.7 × EE
	27	CH_4_ g/day = 49.5 + 12.1 × DMI + 2.57 × NDF
	28	CH_4_ g/day = 136 + 12.3 × DMI − 2.96 × EE
	29	CH_4_ g/day = 49.5 + 12.1 × DMI + 2.57 × NDF
	30	CH_4_ g/day = 279 + 3.53 × NDF
	36	CH_4_ g/day = (13.8 + 0.185 × NDF) × DMI
	37	CH_4_ g/day = (21.8 − 0.452 × EE) × DMI
	38	CH_4_ g/day = (15.4 − 0.354 × EE + 0.173 × NDF) × DMI

**Table 2:** The minimum to maximum enteric methane emissions (g CH_4_/kg DM) values per diet.

	Enteric Methane Emissions (g CH_4_/kg DM)	
Diet	Minimum	Maximum
1	13.45	22.52
2	12.49	22.66
3	13.51	21.98
4	13.15	22.53
5	13.12	23.08
6	13.39	22.68
7	14.43	22.92
8	14.42	22.85
9	14.48	22.96
10	15.08	27.19
11	15.37	28.5
12	15.44	25.92
13	15.25	24.4
14	16.03	30.76
15	16.73	34.27

**Table 3:** The twelve combinations of dietary characteristics that were reviewed, after analysing the correlations between the dietary variables.

Model Number	Variable Combination
1	ME and NDF
2	GE and NDF
3	NDF and EE
4	ME, NDF and FA
5	ME, NDF and EE
6	ME, CP and NDF
7	GE, CP and NDF
8	GE, NDF and FA
9	GE, NDF and EE
10	CP, NDF, FA and EE
11	GE, CP, NDF, FA and EE
12	ME, CP, NDF, FA and EE

**Table 4:** Summary of the 15 UK dairy diets used in the study.

Diet	MEI ^1^	CP (%)	FA (%)	EE (%)	NDF (%)	ADF (%)	Forage (%)
1	222.3	18.5	3	6.4	32.5	26.65	50
2	271.7	17.98	3.56	4.12	32.78	26.88	53.36
3	218.7	22.05	4.5	6.2	33.05	27.1	50
4	262.6	15.6	4.33	4.14	34.87	28.59	69.2
5	268.9	16.05	3.74	4.7	35.15	28.82	50.51
6	255.2	17.43	3.91	4.07	35.47	29.09	57.69
7	210.6	17.4	3.5	6	37.25	30.55	50
8	207	16.2	7.5	5.85	37.5	30.75	50
9	203.4	19.75	4.5	5.65	38.05	31.2	50
10	222.3	19.25	0	5.1	38.85	31.86	50
11	207	16.95	0	4.55	43.85	35.96	50
12	196.2	15.2	2.5	5.45	45	36.9	50
13	202.3	18.08	3.41	4.32	46.11	37.81	81.89
14	136.5	12.58	1.58	2.74	49.16	40.31	73.86
15	125.3	13.97	1.73	3.41	52.5	43.05	81.44

^1^ MEI = (MJ/day).

**Table 5:** The standardized performance of the twelve assessed dietary sequences for the combined enteric prediction equations, including the R^2^, root mean square error (RMSE; g CH_4_/kg DM) and the residuals of variation [29].

Number	Variables		Fixed Effect				Random Error Estimates		Random Effect
			Term	Estimate	Standard Error	t-Value	R^2^	RMSE	Residual Variance
1	ME and NDF		Intercept	19.23	0.42	46.06	0.79	1.47	2.32
		1	NDF	1.88	0.10	19.75 *			
		2	ME	0.31	0.10	3.22 *			
2	GE and NDF		Intercept	19.23	0.42	46.07	0.79	1.47	2.32
		1	NDF	1.88	0.10	19.71 *			
		2	GE	0.31	0.10	3.21 *			
3	NDF and EE		Intercept	19.23	0.42	46.06	0.79	1.48	2.35
		1	NDF	1.76	0.09	20.63 *			
		2	EE	0.16	0.09	1.84			
4	ME, NDF and FA		Intercept	19.23	0.42	46.06	0.80	1.46	2.30
		1	NDF	1.74	0.11	15.18 *			
		2	ME	0.23	0.10	2.27 *			
		3	FA	−0.18	0.08	−2.17 *			
5	ME, NDF and EE		Intercept	19.23	0.42	46.06	0.79	1.47	2.31
		1	NDF	1.88	0.10	19.78 *			
		2	ME	0.44	0.15	2.85 *			
		3	EE	−0.15	0.14	−1.10			
6	ME, CP and NDF		Intercept	19.23	0.42	46.06	0.79	1.47	2.32
		1	NDF	1.87	0.10	19.21 *			
		2	ME	0.32	0.12	2.72 *			
		3	CP	−0.03	0.11	−0.26			
7	GE, CP and NDF		Intercept	19.23	0.42	46.06	0.79	1.47	2.32
		1	NDF	1.87	0.10	19.19 *			
		2	GE	0.32	0.12	2.72 *			
		3	CP	−0.03	0.11	−0.26			
8	GE, NDF and FA		Intercept	19.23	0.42	46.06	0.80	1.46	2.30
		1	NDF	1.74	0.11	15.14 *			
		2	GE	0.23	0.10	2.26 *			
		3	FA	−0.20	0.08	−2.17 *			
9	GE, NDF and EE		Intercept	19.23	0.42	46.06	0.79	1.47	2.31
		1	NDF	1.88	0.10	19.75 *			
		2	GE	0.44	0.15	2.84 *			
		3	EE	−0.15	0.14	−1.09			
10	CP, NDF, FA and EE		Intercept	19.23	0.42	46.06	0.80	1.46	2.30
		1	NDF	1.65	0.10	16.19 *			
		2	FA	−0.28	0.08	−3.32 *			
		3	EE	0.20	0.10	1.99			
		4	CP	−0.00	0.11	−0.02			
11	GE, CP, NDF, FA and EE		Intercept	19.23	0.42	46.06	0.80	1.46	2.30
		1	NDF	1.68	0.14	12.24 *			
		2	FA	−0.24	0.12	−1.98			
		3	GE	0.10	0.25	0.40			
		4	EE	0.13	0.20	0.67			
		5	CP	−0.02	0.11	−0.15			
12	ME, CP, NDF, FA and EE		Intercept	19.23	0.42	46.06	0.80	1.46	2.30
		1	NDF	1.69	0.14	12.31 *			
		2	FA	−0.24	0.12	−1.97			
		3	EE	0.13	0.20	0.65			
		4	ME	0.10	0.25	0.42			
		5	CP	−0.02	0.11	−0.15			

* Asterix highlights a significant t-value (>2 or <−2) for the predictive ability of the dietary component for EMEs within the equation.

## Data Availability

Data on the dietary composition of the example diets is available in the manuscript. Further information on the diets is unavailable due to company confidentiality.

## Abbreviations

The following abbreviations are used in this manuscript:

EME	Enteric methane emissions
EF	Enteric fermentation
CH_4_	Methane
CO_2_	Carbon dioxide
RMSE	Root mean square error
GEI	Gross energy intake
GE	Gross energy
NDF	Neutral detergent fibre
ADF	Acid detergent fibre
EE	Ether extract
ME	Metabolised energy
DMI	Dry matter intake
DM	Dry matter
CP	Crude protein
FA	Fatty acids
GHG	Greenhouse gas
GWP	Global warming potential

## Appendix A

**Table A1:** The correlations between the dietary composition variables, using the 15 diets. The dietary variables consist of GE, ME, CP, FA, EE, NDF, ADF, starch, sugar and DMI.

	GE	ME	CP	FA	EE	NDF	ADF	Starch	Sugar	DMI
GE	1.00 ^1^	1.00	0.77	0.10	0.86	−0.68	−0.68	0.32	0.06	0.09
ME	1.00	1.00	0.77	0.10	0.86	−0.68	−0.68	0.32	0.07	0.09
CP	0.77	0.77	1.00	0.18	0.67	−0.62	−0.62	0.22	0.00	0.17
FA	0.10	0.10	0.18	1.00	0.37	−0.48	−0.48	0.08	−0.15	0.32
EE	0.86	0.86	0.67	0.37	1.00	−0.57	−0.57	0.12	−0.10	−0.05
NDF	−0.68	−0.68	−0.62	−0.48	−0.57	1.00	1.00 ^2^	−0.76	0.19	−0.66
ADF	−0.68	−0.68	−0.62	−0.48	−0.57	1.00	1.00	−0.76	0.19	−0.66
Starch	0.32	0.32	0.22	0.08	0.12	−0.76	−0.76	1.00	−0.43	0.66
Sugar	0.06	0.07	0.00	−0.15	−0.10	0.19	0.19	−0.43	1.00	0.12
DMI	0.09	0.09	0.17	0.32	−0.05	−0.66	−0.66	0.66	0.12	1.00

^1^ ME was derived from GE in the dataset, explaining the high correlation between GE and ME and, hence, the exclusion of GE in the combined model. ^2^ ADF was derived from NDF in the dataset, hence the high correlation between ADF and NDF.

## References

[1] IPCC (1995). Intergovernmental Panel On Climate Change IPCC Second Assessment Climate Change 1995 a Report of the Intergovernmental Panel On Climate Change. https://www.ipcc.ch/site/assets/uploads/2018/02/ipcc_sar_wg_I_full_report.pdf.

[2] Tarighaleslami A.H., Ghannadzadeh A., Atkins M.J., Walmsley M.R. (2020). Environmental life cycle assessment for a cheese production plant towards sustainable energy transition: Natural gas to biomass vs. natural gas to geothermal. J. Clean. Prod..

[3] Mostert P.F., van Middelaar C., Bokkers E., de Boer I. (2018). The impact of subclinical ketosis in dairy cows on greenhouse gas emissions of milk production. J. Clean. Prod..

[4] Dumont B., Groot J.C.J., Tichit M. (2018). Review: Make ruminants green again—How can sustainable intensification and agroecology converge for a better future?. Animal.

[5] Gilardino A., Quispe I., Pacheco M., Bartl K. (2020). Comparison of different methods for consideration of multifunctionality of Peruvian dairy cattle in Life Cycle Assessment. Livest. Sci..

[6] Løvendahl P., Difford G.F., Li B., Chagunda M.G.G., Huhtanen P., Lidauer M.H., Lassen J., Lund P. (2018). Review: Selecting for improved feed efficiency and reduced methane emissions in dairy cattle. Animal.

[7] IPCC (2013). Climate Change 2013: The Physical Science Basis. Contribution of Working Group I to the Fifth Assessment Report of the Intergovernmental Panel on Climate Change.

[8] Pinares-Patiño C.S., Franco F.E., Molano G., Kjestrup H., Sandoval E., MacLean S., Battistotti M., Koolaard J., Laubach J. (2016). Feed intake and methane emissions from cattle grazing pasture sprayed with canola oil. Livest. Sci..

[9] Hammond K.J., Crompton L.A., Bannink A., Dijkstra J., Yáñez-Ruiz D.R., O’Kiely P., Kebreab E., Eugène M., Yu Z., Shingfield K.J. (2016). Review of current in vivo measurement techniques for quantifying enteric methane emission from ruminants. Anim. Feed Sci. Technol..

[10] Hristov A.N., Kebreab E., Niu M., Oh J., Bannink A., Bayat A., Boland T., Brito A., Casper D., Crompton L. (2018). Symposium review: Uncertainties in enteric methane inventories, measurement techniques, and prediction models. J. Dairy Sci..

[11] Patra A.K. (2016). Recent Advances in Measurement and Dietary Mitigation of Enteric Methane Emissions in Ruminants. Front. Vet. Sci..

[12] Benaouda M., Martin C., Li X., Kebreab E., Hristov A.N., Yu Z., Yáñez-Ruiz D.R., Reynolds C.K., Crompton L.A., Dijkstra J. (2019). Evaluation of the performance of existing mathematical models predicting enteric methane emissions from ruminants: Animal categories and dietary mitigation strategies. Anim. Feed Sci. Technol..

[13] IPCC (1996). Revised 1996 IPCC Guidelines for National Greenhouse Gas Inventories: Workbook, Chapter 4—Agriculture (Enteric Fermentation). https://www.ipcc-nggip.iges.or.jp/public/gp/bgp/4_1_CH4_Enteric_Fermentation.pdf.

[14] IPCC (2006). Chapter 10 Emissions from Livestock and Manure Management. https://www.ipcc-nggip.iges.or.jp/public/2006gl/.

[15] Ouatahar L., Bannink A., Lanigan G., Amon B. (2021). Modelling the effect of feeding management on greenhouse gas and nitrogen emissions in cattle farming systems. Sci. Total Environ..

[16] Vibart R., de Klein C., Jonker A., van der Weerden T., Bannink A., Bayat A.R., Crompton L., Durand A., Eugène M., Klumpp K. (2021). Challenges and opportunities to capture dietary effects in on-farm greenhouse gas emissions models of ruminant systems. Sci. Total Environ..

[17] Rotz C.A., Montes F., Chianese D.S. (2010). The carbon footprint of dairy production systems through partial life cycle assessment. J. Dairy Sci..

[18] Patra A.K. (2013). The effect of dietary fats on methane emissions, and its other effects on digestibility, rumen fermentation and lactation performance in cattle: A meta-analysis. Livest. Sci..

[19] Jose V.S., Sejian V., Bagath M., Ratnakaran A.P., Lees A.M., Al-Hosni Y.A.S., Sullivan M., Bhatta R., Gaughan J.B. (2016). Modeling of Greenhouse Gas Emission from Livestock. Front. Environ. Sci..

[20] Niu M., Appuhamy J.A.D.R.N., Leytem A.B., Dungan R.S., Kebreab E. (2016). Effect of dietary crude protein and forage contents on enteric methane emissions and nitrogen excretion from dairy cows simultaneously. Anim. Prod. Sci..

[21] Grandl F., Furger M., Kreuzer M., Zehetmeier M. (2019). Impact of longevity on greenhouse gas emissions and profitability of individual dairy cows analysed with different system boundaries. Animal.

[22] van Gastelen S., Dijkstra J., Bannink A. (2019). Are dietary strategies to mitigate enteric methane emission equally effective across dairy cattle, beef cattle, and sheep?. J. Dairy Sci..

[23] Børsting C.F., Brask M., Hellwing A., Weisbjerg M., Lund P. (2020). Enteric methane emission and digestion in dairy cows fed wheat or molasses. J. Dairy Sci..

[24] Arndt C., Hristov A.N., Price W.J., McClelland S.C., Pelaez A.M., Cueva S.F., Oh J., Bannink A., Bayat A.R., Crompton L.A. (2021). Strategies to Mitigate Enteric Methane Emissions by Ruminants—A Way to Approach the 2.0 °C Target View project Impact of CFI methodologies on whole-farm systems View project. AgriRxiv.

[25] Appuhamy J.A.D.R.N., France J., Kebreab E. (2016). Models for predicting enteric methane emissions from dairy cows in North America, Europe, and Australia and New Zealand. Glob. Change Biol..

[26] R Core Team (2022). The R Project for Statistical Computing. https://www.r-project.org/.

[27] De Haas Y., Windig J., Calus M., Dijkstra J., de Haan M., Bannink A., Veerkamp R. (2011). Genetic parameters for predicted methane production and potential for reducing enteric emissions through genomic selection. J. Dairy Sci..

[28] Niu M., Kebreab E., Hristov A.N., Oh J., Arndt C., Bannink A., Bayat A.R., Brito A.F., Boland T., Casper D. (2018). Prediction of enteric methane production, yield, and intensity in dairy cattle using an intercontinental database. Glob. Change Biol..

[29] Baker F., O’Grady L., Green M. (2024). Final Report Student Project No. 41140078/41240004. https://projectblue.blob.core.windows.net/media/Default/Dairy/41240004PhDModellingCEmissionsDairyFINAL.pdf.

[30] Ellis J.L., Kebreab E., Odongo N., McBride B., Okine E., France J. (2007). Prediction of methane production from dairy and beef cattle. J. Dairy Sci..

[31] Van Lingen H.J., Fadel J.G., Bannink A., Dijkstra J., Tricarico J.M., Pacheco D., Casper D.P., Kebreab E. (2018). Multi-criteria evaluation of dairy cattle feed resources and animal characteristics for nutritive and environmental impacts. Animal.

[32] Moate P.J., Williams S., Grainger C., Hannah M., Ponnampalam E., Eckard R. (2011). Influence of cold-pressed canola, brewers grains and hominy meal as dietary supplements suitable for reducing enteric methane emissions from lactating dairy cows. Anim. Feed Sci. Technol..

[33] Nielsen N.I., Volden H., Åkerlind M., Brask M., Hellwing A.L.F., Storlien T., Bertilsson J. (2013). A prediction equation for enteric methane emission from dairy cows for use in NorFor. Acta Agric. Scand. Sect. A.

[34] Thomas C. (2004). Feed into Milk: A New Applied Feeding System for Dairy Cows: An Advisory Manual.

[35] Gelman A., Hill J. (2007). Data Analysis Using Regression and Multilevel/Hierarchical Models.

[36] Johnston R., Jones K., Manley D. (2018). Confounding and collinearity in regression analysis: A cautionary tale and an alternative procedure, illustrated by studies of British voting behaviour. Qual. Quant..

